# A Global Look at Laser Tattoo Removal Research: Key Areas and Emerging Trends Revealed Through Bibliometric and Visualization Analysis

**DOI:** 10.1111/jocd.16748

**Published:** 2024-12-27

**Authors:** Sa'ed H. Zyoud

**Affiliations:** ^1^ Department of Clinical and Community Pharmacy, College of Medicine and Health Sciences An‐Najah National University Nablus Palestine; ^2^ Clinical Research Centre An‐Najah National University Hospital Nablus Palestine

To the editor,

The article “Retrospective clinical evaluation of Q‐switched Nd:YAG laser safety and efficacy in tattoo removal: A new perspective on the Kirby‐Desai scale” by Egozi and Toledano [1] in the Journal of Cosmetic Dermatology caught my attention. The study highlights the Q‐switched Nd:YAG laser as a safe and effective method for tattoo removal, requiring fewer treatments than previously thought. Surprisingly, despite the significance of this topic, I couldn't locate any bibliometric analyses on laser tattoo removal in my search across various databases.

In recent years, bibliometric analysis has gained importance as a means of assessing research boundaries and locating new concepts in a variety of fields, including dermatology. A thorough bibliometric analysis of laser tattoo removal research is still lacking, despite the value of the methodology. The aim of this paper is to overcome this gap by conducting a comprehensive global bibliographic analysis of laser tattoo removal. Its goal is to raise the evaluate for laser tattoo removal research in general, encourage researchers to consider new research topics, and provide a comprehensive overview of the knowledge structure and regular trends in the field.

Research activities on laser tattoo removal were examined through bibliometric analysis, focusing on identifying the main contributors and highlighting recurring themes in this field. Using a comprehensive cross‐sectional bibliometric methodology, we extracted documents from the Scopus database covering the period before December 31, 2023. The search was conducted using keywords related to “removal of laser tattoos.” The researchers used VOSviewer (version 1.6.20) software to visually represent repeated terms and themes [2]. Research has ignored other types of records in favor of focusing solely on publications from scientific journals.

A worldwide search from 1865 to 2023 revealed a remarkable set of 3899 documents with titles primarily addressing tattoo‐related topics. Between 1978 and 2023, 318 scientific articles on the use of lasers for tattoo removal were published. Of the collected papers in this group, 251 (78.93%) were considered original. They were followed by 29 reviews (9.12%), 25 letters (7.86%), and 13 other types of articles (4.09%), such as notes, editorials, and conference abstracts. English was the most widely used language in the respective publications, with 284 entries. The next most commonly used languages are German and French, with 14 and 10 entries, respectively. A total of 96.86% of all relevant publications were written in one of these three languages.

Figure 1 illustrates the distribution of publications concerning laser tattoo removal. Over the period from 1978 to 2023, a modest yet fluctuating increase in such publications was observed (*R* ^2^ = 0.75; *p* value < 0.001). The growth and productivity trends in publications related to laser tattoo removal have been shaped by developments in medical research, advancements in laser technology, and clinical practice [3, 4]. These factors collectively contribute to the advancement of our understanding of the condition, improvement in treatment methodologies, and the establishment of standardized findings for clinical studies. Table 1 presents the top 10 countries contributing to publications on laser tattoo removal. These countries included the United States (*n* = 130; 40.88%), Germany (*n* = 27; 8.49%), the United Kingdom (*n* = 20; 6.29%), and Italy (*n* = 14; 4.40%).

**FIGURE 1:**
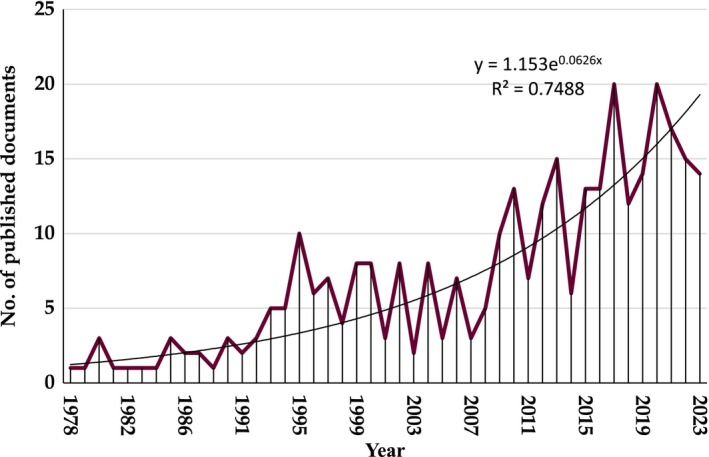
Annual growth of published research related to laser tattoo removal (1978–2023).

**TABLE 1:** Ten leading countries in publications concerning laser tattoo removal.

Ranking	Country	No. of documents	%
1st	United States	130	40.88
2nd	Germany	27	8.49
3rd	United Kingdom	20	6.29
4th	Italy	14	4.40
5th	France	13	4.09
5th	South Korea	13	4.09
7th	Japan	12	3.77
8th	China	11	3.46
9th	Switzerland	9	2.83
10th	Brazil	7	2.20
10th	India	7	2.20
10th	Spain	7	2.20

This study utilized VOSviewer 1.6.20 to construct a term co‐occurrence map based on keywords appearing at least 15 times in titles and abstracts (binary counting). The map prioritizes terms with high relevance scores, ensuring prominent visualization for frequently co‐occurring terms (large bubbles) and those with high similarity (close proximity). As shown in Figure 2A, larger circles represent frequently occurring terms. Three main thematic clusters emerged:

1. Effectiveness of Laser Type (Green Cluster): This cluster focuses on the comparative efficacy of Q‐switched ruby and neodymium Nd:YAG lasers for blue and black tattoo removal. The findings suggest that the Nd:YAG laser offers greater efficiency and requires fewer removal sessions.
2. Complications of Laser Tattoo Removal (Blue Cluster): While generally safe, laser tattoo removal can lead to complications such as hyperpigmentation, hypopigmentation, ink darkening, and scarring.
3. Advancements in Laser Technology (Red Cluster): This cluster explores the application of picosecond lasers for safe and effective tattoo removal, including red and yellow pigments.
  After 2014, a shift emerged toward the use of picosecond lasers in laser tattoo removal research (Figure 2B). This is likely due to their shorter pulse durations, which lead to faster heating of the targeted pigments (chromophores) and, consequently, more efficient tattoo removal [5].
  In conclusion, research on laser tattoo removal has increased recently, particularly in the past 10 years. This study presents a wide range of academic research on laser tattoo removal, with global participation and a consistent growth trend. This review highlights developments in clinical practice and laser technology, which have enhanced treatment results and standardized conclusions. Since 2014, there has been a notable shift in research focus toward more effective tattoo removal techniques, as evidenced by the development of picosecond lasers. This succinct analysis highlights the dynamic evolution of laser tattoo removal and its critical role in improving our knowledge and oversight of this practice. The current surge in research has significant effects on clinical practice. By staying up to date on new developments in their fields of interest and trends, clinicians can enhance patient care. Furthermore, a thorough examination of earlier research can reveal knowledge gaps, focusing future investigations on the most crucial problems. Ultimately, a more thorough understanding of the corpus of research may lead to improved clinical judgment grounded in best practices, potentially improving patient outcomes and advancing the dermatological field.

**FIGURE 2:**
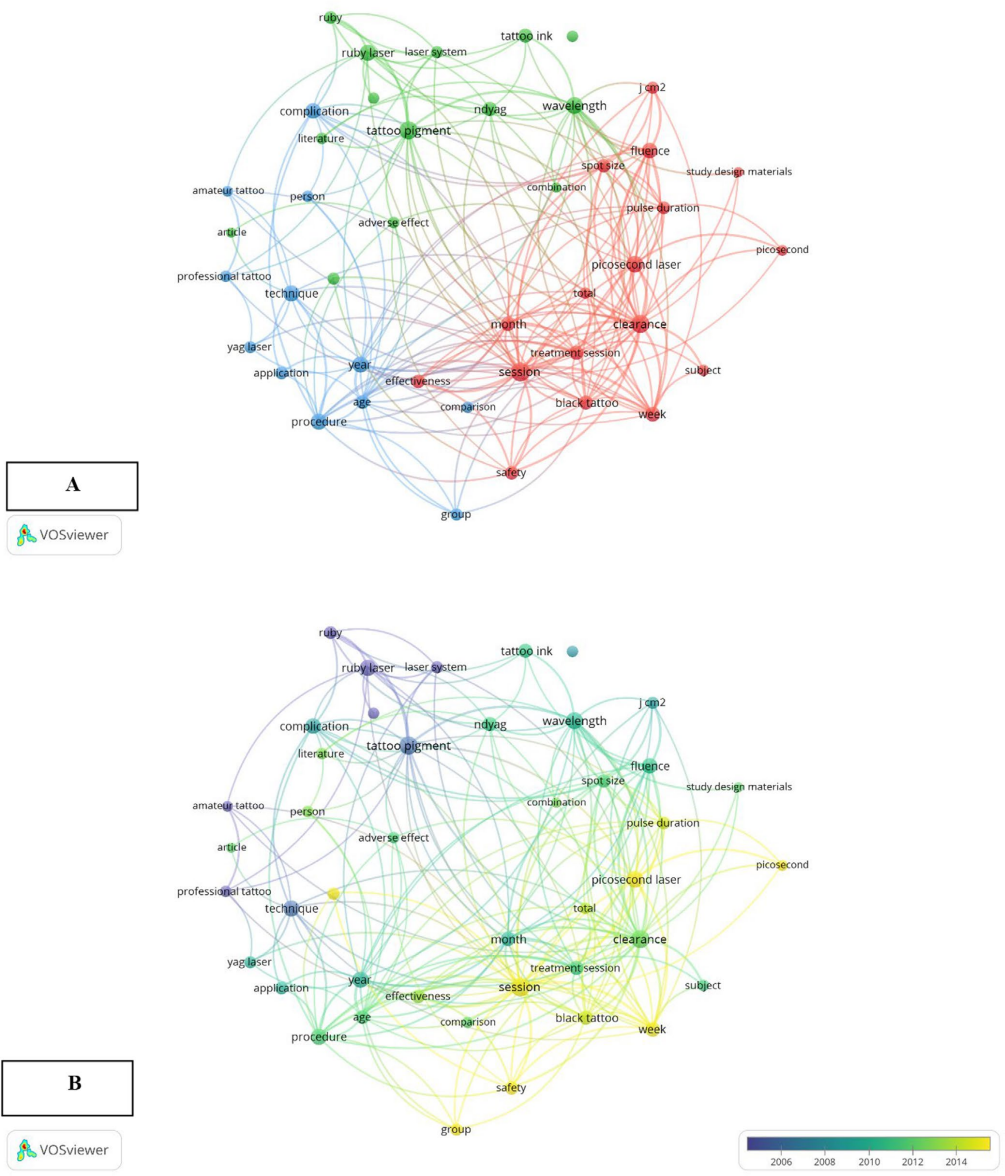
Mapping of terms used in research on laser tattoo removal. (A) The co‐occurrence network of terms extracted from the title or abstract of at least 40 articles. The colors represent groups of terms that are relatively strongly linked to each other. The size of a term signifies the number of publications related to laser tattoo removal in which the term appeared, and the distance between two terms represents an estimated indication of the relatedness of these terms. (B) Mapping of terms used in research on laser tattoo removal. The terms “early” (blue) or “late” (yellow) years indicate when the term appeared.

## Author Contributions

Sa'ed H. Zyoud significantly contributed to the conceptualization and design of the research project, overseeing data management and analysis, generating figures, and making substantial contributions to the literature search and interpretation. Furthermore, Sa'ed H. Zyoud authored the manuscript, which he reviewed and approved as the sole author.

## Ethics Statement

The author has nothing to report.

## Consent

The author has nothing to report.

## Conflicts of Interest

The author declares no conflicts of interest.

## Data Availability

This published article contains all the information produced or examined in this research. Additional datasets utilized during this study can be obtained from the corresponding author.
